# Supplementary material for: Effect of Significant Coronary Artery Stenosis on Prognosis in Patients with Vasospastic Angina: A Propensity Score-Matched Analysis
Source: J Clin Med. 2021 Jul 28;10(15):3341. doi: 10.3390/jcm10153341 (PMC8347544; doi:10.3390/jcm10153341)
Supplement: Supplementary file 1 [file jcm-10-03341-s001.zip › jcm-1319169-supplementary.pdf]

Supplementary Table S1. One-vessel vs. Multi-vessel disease

**One-year**	**Significant stenosis (*n* = 189)**	**One-vessel (*n* = 161)**	**Multi-vessel (*n*= 28)**	***p*-value**
Composite events	11 (5.8)	8 (5.0)	3 (10.7)	0.211
ACS	9 (4.8)	7 (4.3)	2 (7.1)	0.624
Cardiac death	0 (0.0)	0 (0.0)	0 (0.0)	-
VT or VF	1 (0.5)	0 (0.0)	1 (3.6)	0.148
AV block	1 (0.5)	1 (0.6)	0 (0.0)	1.000
All-cause death	0 (0.0)	0 (0.0)	0 (0.0)	-
**Total period**				
Composite events	17 (9.0)	14 (8.7)	3 (10.7)	0.722
ACS	14 (7.4)	12 (7.5)	2 (7.1)	1.000
Cardiac death	0 (0.0)	0 (0.0)	0 (0.0)	-
VT or VF	2 (1.1)	1 (0.6)	1 (3.6)	0.276
AV block	1 (0.5)	1 (0.6)	0 (0.0)	1.000
All-cause death	0 (0.0)	0 (0.0)	0 (0.0)	-

ACS, acute coronary syndrome; AV, atrioventricular; VA, vasospastic angina; VF, ventricular fibrillation; VT, ventricular tachycardia

Supplementary Table S2. Baseline characteristics of matched population

	All (n = 546)	No significant stenosis (n =364)	Significant stenosis (n = 182)	p-value
Age, years	58.2 ± 11.2	57.9 ± 11.4	58.8 ± 10.6	0.368
Male, n (%)	417 (76.4)	282 (77.5)	135 (74.2)	0.393
BMI, kg/m^2	24.7 ± 3.6	24.8 ± 3.5	24.7 ± 3.8	0.767
SBP, mmHg	128.0 ± 18.7	128.4 ± 18.2	127.1 ± 19.8	0.439
DBP, mmHg	76.7 ± 12.4	76.9 ± 11.5	76.1 ± 14.0	0.448
Previous CAD, n (%)	85 (15.6)	53 (14.6)	32 (17.6)	0.359
Diabetes mellitus, n (%)	102 (18.7)	68 (18.7)	34 (18.7)	1.000
Hypertension, n (%)	280 (51.3)	187 (51.4)	93 (51.1)	0.952
Dyslipidemia, n (%)	88 (16.1)	59 (16.2)	29 (15.9)	0.934
Alcohol drinking, n (%)	287 (52.6)	195 (53.6)	92 (50.5)	0.505
Current smoking, n (%)	210 (38.5)	146 (40.1)	64 (35.2)	0.263
**Laboratory finding**				
Hemoglobin, g/dL	14.0 ± 1.7	14.0 ± 1.8	13.9 ± 1.5	0.631
Creatinine, mg/dL	0.8 ± 0.2	0.8 ± 0.2	0.8 ± 0.2	0.207
Glucose, mg/dL	115.1 ± 37.9	115.9 ± 40.6	113.5 ± 32.2	0.501
hs-CRP, mg/dL	1.0 ± 6.1	1.1± 7.1	0.7 ± 3.2	0.459
Total cholesterol, mg/dL	169.2 ± 37.3	169.4 ± 36.6	168.8 ± 38.8	0.879
LDL cholesterol, mg/dL	99.3 ± 32.6	99.4 ± 32.0	99.1 ± 33.7	0.934
Triglyceride, mg/dL	150.5 ± 104.7	147.3 ± 99.5	157.0 ± 114.4	0.342
HDL cholesterol, mg/dL	45.1 ± 11.9	45.6 ± 12.2	44.2 ± 11.3	0.212
LV EF, %	63.9 ± 7.0	64.2 ± 6.6	63.4 ± 7.8	0.265
**Clinical diagnosis before ergonovine**				
Angina, n (%)	491 (89.9)	330 (90.7)	161 (88.5)	0.421
Myocardial infarction, n (%)	10 (1.8)	4 (1.1)	6 (3.3)	0.091
Cardiac arrest, n (%)	8 (1.5)	6 (1.6)	2 (1.1)	0.725
Syncope, n (%)	6 (1.1)	4 (1.1)	2 (1.1)	1.000
VT or VF, n (%)	4 (0.7)	3 (0.8)	1 (0.5)	1.000
AV block, n (%)	0 (0.0)	0 (0.0)	0 (0.0)	-

AV, atrioventricular; BMI, body mass index; CAD, coronary artery disease; CCB, calcium-channel blocker; DBP, diastolic blood pressure; HDL, high-density lipoprotein; hs-CRP, high

sensitive-C reactive protein; LDL, low-density lipoprotein; LV EF, left ventricular ejection fraction; SBP, systolic blood pressure; VF, ventricular fibrillation; VT, ventricular tachycardia.
